# Supplementary figures and images for: Rapid drop in the reproduction number during the Ebola outbreak in the Democratic Republic of Congo
Source: PeerJ. 2015 Nov 19;3:e1418. doi: 10.7717/peerj.1418 (PMC4655090; doi:10.7717/peerj.1418)

Case reproduction number  $R$

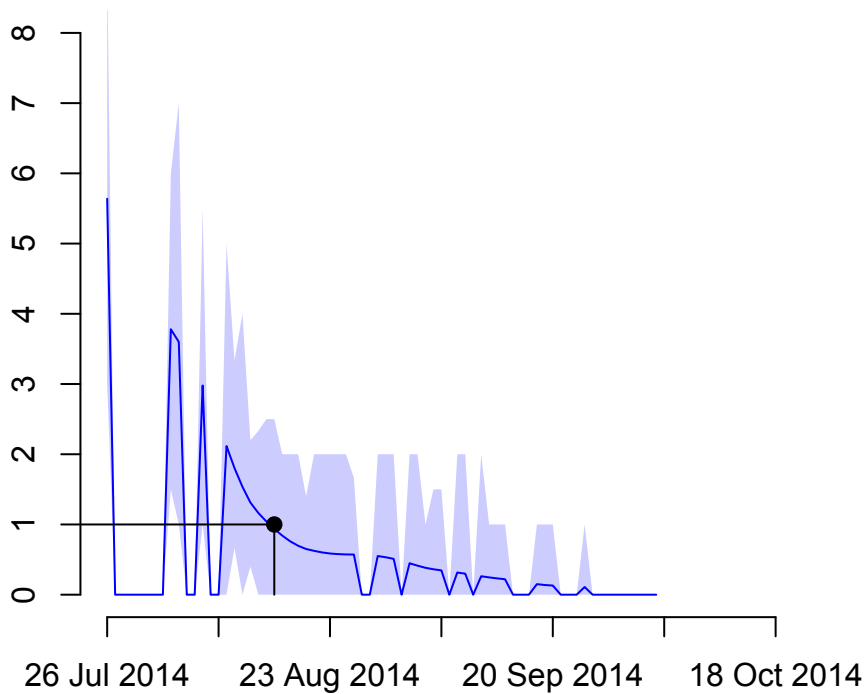

Supplement: Figure S1 — The blue shaded area represents the 95% confidence interval of the estimates. The black dot denotes the time at which R dropped below unity. [file peerj-03-1418-s004.pdf]
